# Increase in Sialylation and Branching in the Mouse Serum *N*-glycome Correlates with Inflammation and Ovarian Tumour Progression

**DOI:** 10.1371/journal.pone.0071159

**Published:** 2013-08-30

**Authors:** Radka Saldova, Helene Piccard, Marta Pérez-Garay, David J. Harvey, Weston B. Struwe, Marie C. Galligan, Nele Berghmans, Stephen F. Madden, Rosa Peracaula, Ghislain Opdenakker, Pauline M. Rudd

**Affiliations:** 1 National Institute for Bioprocessing Research and Training (NIBRT) GlycoScience Group, Dublin, Ireland; 2 Laboratory of Immunobiology, Rega Institute for Medical Research, University of Leuven, Leuven, Belgium; 3 Unitat de Bioquímica i Biologia Molecular, Departament de Biologia, Universitat de Girona, Girona, Spain; 4 Oxford Glycobiology Institute, Department of Biochemistry, University of Oxford, Oxford, United Kingdom; 5 School of Mathematical Sciences, University College Dublin, Dublin, Ireland; 6 National Institute for Cellular Biotechnology, Dublin City University, Dublin, Ireland; National Institutes of Health, United States of America

## Abstract

Ovarian cancer is the most lethal gynaecological cancer and is often diagnosed in late stage, often as the result of the unavailability of sufficiently sensitive biomarkers for early detection, tumour progression and tumour-associated inflammation. Glycosylation is the most common posttranslational modification of proteins; it is altered in cancer and therefore is a potential source of biomarkers. We investigated the quantitative and qualitative effects of anti-inflammatory (acetylsalicylic acid) and pro-inflammatory (thioglycolate and chlorite-oxidized oxyamylose) drugs on glycosylation in mouse cancer serum. A significant increase in sialylation and branching of glycans in mice treated with an inflammation-inducing compound was observed. Moreover, the increases in sialylation correlated with increased tumour sizes. Increases in sialylation and branching were consistent with increased expression of sialyltransferases and the branching enzyme MGAT5. Because the sialyltransferases are highly conserved among species, the described changes in the ovarian cancer mouse model are relevant to humans and serum *N*-glycome analysis for monitoring disease treatment and progression might be a useful biomarker.

## Introduction

Ovarian cancer is the fifth most common cancer in females and the second most common gynaecological cancer. Although it is the most lethal of all gynaecological cancers among women in Europe [1], early diagnosis can considerably prolong life expectancy and quality of life. However, most patients are diagnosed when the disease is in an advanced stage [2]. The 5-year survival rate, which is more than 90% for early stage diagnosis, decreases in advanced stages to only about 30% [2]. Currently ovarian cancer patients are subjected to CT scans and CA125 levels to monitor disease recurrence or progression, but these tests are frequently not sensitive and specific enough to detect the cancer in early stages [3]. More sensitive tests, such as the presence of cancer-specific serum biomarkers are urgently needed and to this end we have investigated the use of specific *N*-glycans from serum. Changes in glycosylation are found in many cancers and inflammatory conditions such as acute and chronic inflammatory diseases (sepsis, pancreatitis, rheumatoid arthritis, diabetes) and infection [4]. The most common glycosylation alterations to *N*-glycans in cancer and chronic inflammatory conditions are increases in sialylation, branching and fucosylation [5], [6].

Inflammation and infection increase the risk of ovarian cancer [7]. Inflammation has been found to be one of the critical processes that govern cancer biology following experiments showing that inflammatory cells, cytokines and chemokines contribute to tumour growth and progression [8], [9]. Consequently, non-steroidal anti-inflammatory drugs reduce the risk of cancer, decrease cell growth, induce apoptosis and inhibit the survival of cancer cells [7], [10], [11]. Chlorite-oxidized oxyamylose (COAM) is an antiviral agent effective in the treatment of virus-induced cancer and in a model of mammary cancer in C3H mice [12], [13]. Recently, it was demonstrated that COAM is a pro-inflammatory drug, inducing and binding chemokines [14], [15].

The aim of this study was to investigate whether changes occur in *N*-glycosylation with anti- and pro-inflammatory drug treatments in a tumour-bearing host, whether a correlation exists with tumour size and whether this can be analyzed in serum samples as a useful biomarker. We have based our study on the mouse experimental model in which tumour size was found to be increased with pro- and decreased with anti-inflammatory drugs and in which macrophages had a significant influence on tumour progression [16]. We have analysed mouse serum samples using our fully quantitative high-throughput *N*-glycan analysis based on high performance liquid chromatography (HPLC)-separation of glycans released from the serum.

## Materials and Methods

### Reagents

COAM was synthesized by a two-step oxidation of amylose by the method described by Claes *et al.* [17], analyzed for endotoxin contamination by the *Limulus* amebocyte lysate test (Cambrex Bio Science, Verviers, Belgium) and quality-controlled as described [18]. COAM was dissolved in phosphate-buffered saline (PBS) and stored at −20°C. For working solutions, the PBS stocks were diluted in endotoxin-free saline (0.9% NaCl, Baxter, Braine-l’Alleud, Belgium) to the desired concentration.

### Xenogeneic ovarian carcinoma model

OVCAR3 adenocarcinoma cells (ATCC Number HTB-161) were grown as monolayer cultures in stationary flasks (75 cm^2^) in RPMI 1640 medium (Lonza, Verviers, Belgium) with 4 mM glutamine, supplemented with 10%(v/v) heat-inactivated foetal calf serum (FCS) and penicillin/streptomycin at 37°C in an atmosphere of 5%CO_2_. The animal experiments were approved by the ethical committee of the University of Leuven. The experimental ovarian carcinoma tumour model [16] involved the resuspension of 1×10^7^ OVCAR3 cells in PBS/Matrigel (1∶1) in a total volume of 0.5 mL. This suspension was injected on day 0 into the peritoneum of female C.B-17/lcr severe combined immunodeficient (scid/scid) mice (8–12 weeks) which were bred and housed under specific pathogen-free conditions. The injected mice were divided into different groups for each experiment. One group (n = 5) was treated with acetylsalicylic acid (ASA, 100 mg/kg in PBS; pH5.0) by daily s.c. injection, one group (n = 5) with thioglycolate (TG) broth (0.5 mL of a 3% solution, Fluka-Sigma-Aldrich, Bornem, Belgium) by i.p. treatment twice per week, and one group (n = 5) with COAM (2 mg in PBS, pH neutral) by i.p. injection once per week. Control mice were administered PBS s.c. (pH 5.0, daily, n = 5) or i.p. (neutral pH, twice per week, n = 5). A separate group (n = 5) was injected with tumour cells but maintained without a weekly peritoneal lavage procedure. An additional group of mice (n = 4) did not receive tumour cells, nor peritoneal lavage. The weekly peritoneal lavages comprised the injection of 4 mL of ice-cold PBS (with 2% FCS and 20 U/mL heparin) into the peritoneum of anaesthetized mice. The peritoneum was gently massaged during 1 min, and the fluid was retrieved and pooled per treatment group. Seven weeks after OVCAR3 cell injection, all mice were sacrificed and the tumour foci were collected and counted. The tumour diameters were measured by use of a caliper. The tumour volumes were evaluated with the formula (4πab^2^)/3, with a and b the largest and smallest radii, respectively. Blood was collected from individual mice by retro-orbital puncture and incubated for 1 h at room temperature, followed by 6 h at 4°C to obtain serum. Serum was collected upon two centrifugations of 8 min at 1100 rpm and stored at −80°C until glycan analyses. The *in vivo* experiment was performed twice.

### Flow cytometry analysis

Flow cytometry analysis was performed weekly to characterize peritoneal cell populations. Peritoneal cell suspensions were centrifuged and the collected cells were resuspended and erythrocytes were lysed in lysis buffer (0.15 M NH_4_Cl, 1.0 mM KHCO_3_, 0.1 mM Na_2_EDTA, pH 7.2, 3 min incubation, 37°C). The lysis process was stopped by addition of fluorescent-activated cell sorting (FACS) buffer (PBS with 2%FCS). The remaining cells were washed twice and resuspended. Cell numbers were counted. Single-cell suspensions (0.12–0.5×10^6^ living cells) were passed through nylon meshes (tubes with cell-strainer cap, BD Falcon, Erembodegem, Belgium), washed in 500 µL FACS buffer and incubated for 15 min with Fc receptor-blocking antibodies (0.5 µL/0.5×10^6^ cells; anti-mouse CD16/CD32; Miltenyi Biotec, Bergisch Gladbach, Germany). After a wash step with FACS buffer, cells were stained for markers of different innate immune cell types with the indicated fluorophore-conjugated antibodies (eBioscience, San Diego, CA) during minimally 20 min. Washed cells were fixed with 0.4%formaldehyde in PBS. Alternatively, for staining of apoptotic and necrotic cells, the Annexin-V-FLUOS Staining Kit (Roche Applied Science, Vilvoorde, Belgium) was applied on non-fixed cells. Cells were analyzed on a FACSCalibur flow cytometer (between 5,000 and 20,000 events being acquired) and data were processed with CellQuest software (Becton Dickinson Immunocytometry Systems, San Jose, CA).

### *N*-glycan analysis

*N*-glycans were released from 5 µl sera using a high-throughput method [19]. Briefly, samples were reduced and alkylated in 96-well plates, set into SDS-gel blocks and washed. The *N*-linked glycans were released using peptide *N*-glycanase F (1000 U/ml; EC3.5.1.52) [20], [21] and labelled by reductive amination with the 2-aminobenzamide [20] (Ludger Tag™ 2-AB glycan labelling kit, Ludger Ltd, Oxford, UK).

The 2-AB-labelled glycans were digested in 10 µL of 50 mM sodium acetate buffer, pH5.5 for 18 h at 37°C, using arrays of the following enzymes (Prozyme, San Leandro, CA, USA) at the indicated concentrations: *Arthrobacter ureafaciens* sialidase (EC3.2.1.18), 1 U/mL; *Streptococcus pneumoniae* sialidase (EC3.2.1.18), 1 U/mL; coffee bean α-galactosidase (EC3.2.1.22), 25 U/ml; bovine testes β-galactosidase (EC3.2.1.23), 1 U/mL; *Streptococcus pneumoniae* β-galactosidase (EC3.2.1.23), 0.1 U/mL; jack bean β-*N-*acetylhexosaminidase (EC3.2.1.24), 50 U/mL; bovine kidney α-fucosidase (EC3.2.1.51), 1 U/mL; almond meal α-fucosidase (EC3.2.1.111), 3 mU/mL; *Xanthomonus sp.* α-fucosidase (EC3.2.1.51.), 0.1 U/ml; jack bean α-mannosidase (EC3.2.1.24), 50 U/mL and β-*N*-acetylglucosaminidase cloned from *Streptococcus pneumonia,* expressed in *E. coli* (EC 3.2.1.30), 4 U/mL. After incubation, enzymes were removed by filtration through protein-binding EZ filters (Millipore Corporation) [22].

Hydrophilic interaction liquid chromatography (HILIC) HPLC was performed using a TSK-Gel Amide-80 4.6×250 mm column (Anachem, Luton, Bedfordshire, UK) on a 2695 Alliance separations module (Waters, Milford, MA) and a Waters 2475 fluorescence detector. Solvent A was 50 mM formic acid adjusted to pH4.4 with ammonia solution. Solvent B was acetonitrile. The column temperature was set to 30?C. The 60 minutes high-throughput method was used: a linear gradient of 35–47% solvent A over 48 min at a flow rate of 0.8 mL/min, followed by 1 min at 47–100%A and 4 min at 100%A, returning to 35%A over 1 min and then finishing with 35%A for 6 min [19]. Samples were injected in 80% acetonitrile [22]. The system was calibrated using an external standard of hydrolyzed and 2-aminobenzamide-labelled glucose oligomers to create a dextran ladder [22].

Weak anion exchange (WAX)-HPLC was performed using a Vydac 301VHP575 7.5×50-mm column (Anachem) on a 2695 Alliance separations module with a Waters 474 fluorescence detector. Solvent A was 0.5 M formic acid adjusted to pH9.0 with ammonia solution, and solvent B was 10% (v/v) methanol in water. Gradient conditions were as follows: a linear gradient of 0–5%A over 12 min at a flow rate of 1 mL/min, followed by 5–21%A over 13 min and then 21–50% A over 25 min, 80–100% A over 5 min, and then 5 min at 100%A. Samples were injected in water. A fetuin *N*-glycan standard was used for calibration [22].

### Sialic acid analysis

Sialic acids were released and derivatized with 1,2-diamino-4,5-methylenedioxybenzene according to the manufacturer’s instructions (Ludger, Abingdon, UK) [23]. The labelled samples were analysed using reversed phase HPLC on a Waters XBridge BEH C18 150×2.1 mm i.d., 3.5 µm column with fluorescence detection.

### Electrospray (ESI) mass spectrometry

*N*-glycans were released from the glycoproteins with PNGase F as described above and cleaned with a Nafion membrane [24]. Samples were dissolved in water:methanol (1∶1, v:v) containing 0.1 M ammonium phosphate and infused into the nanospray ion source of a Waters quadrupole-time-of-flight Ultima Global instrument (Waters, Manchester, UK) using Proxeon nanospray capillaries (Thermo Fisher Scientific, UK) The ion source conditions were: temperature, 120°C; nitrogen flow 50 L/hr; infusion needle potential, 1.2 kV; cone voltage 100 V; RF-1 voltage 150 V. Negative ion mass spectrometry (MS) and collision-induced dissociation spectra (2 sec scans) were acquired with a digitization rate of 4 GHz and accumulated until a satisfactory signal:noise ratio had been obtained. For MS/MS data acquisition, the parent ion was selected at low resolution (about 4 m/z mass window) to allow transmission of isotope peaks and fragmented with argon. The voltage on the collision cell was adjusted with mass and charge to give an even distribution of fragment ions across the mass scale. Typical values were 80–120 V. Other voltages were as recommended by the manufacturer. Instrument control, data acquisition and processing were performed with MassLynx software Version 4.0. Ions were detected as phosphate adducts and spectral interpretation was as described in reference [25]. Desialylation of the *N*-glycans for mass spectrometric analysis was performed by heating 1 µL of an aqueous solution with 1 µL of 1% acetic acid for 30 mins at 80°C.

### Quantitative PCR (qPCR)

Total RNA from mouse livers was extracted using the RNeasy® RNA isolation kit (Qiagen, Hilden, Germany) including on-column DNase digestion. RNA yield and purity were determined spectrophotometrically and by denaturing agarose gel electrophoresis, respectively. Single-stranded cDNA was synthesised from 1.5 µg of total RNA using the High-Capacity cDNA Reverse Transcription Kit (Applied Biosystems Inc, Foster City, CA). Primers and probe sequences for the endogenous gene TBP (reference Mm00446973_m1*) and the genes ST3Gal1 (Mm00501493_m1*), ST3Gal3 (Mm00493353_m1*), ST3Gal4 (Mm00501503_m1*), ST3Gal6 (Mm00450665_m1*), ST6Gal1 (Mm00486119_m1*), MGAT5 (Mm00455036_m1*) were pre-designed TaqMan™ Gene Expression Assays from Applied Biosystems-Applera Hispania SA, Spain. All PCRs reactions were performed in optical 96-well plates with an ABI PRISM 7300 Sequence Detector System in a total volume of 20 µl containing 9 µl of cDNA diluted in RNAse free water, 10 µl of TaqMan® Universal Master Mix No AmpErase® and 1 µl of the corresponding Custom Taqman Gene Expression Assay™. The following standard thermal profile was used for all PCRs: 95°C for 10 min; 40 cycles of 95°C for 15 s and 60°C for 1 min and data were analysed with 7300 SDS 1.3.1 software (Applied Biosystems). The relative concentrations of the genes were calculated by the comparative Ct Method (ΔΔCts). TBP was used as a reference gene to normalize the results. The data for each mouse comprises the mean±SD of four replicates. The data for each mouse group corresponds to the mean±SD of the data for the four mice in that group (N = 16). Normality of data (x) was tested using the Kolmogorov-Smirnov test and the homogeneity of variances was checked using the Levene’s test. The differences between data (all with normal distribution and homogenous variances) were analysed using the parametric Student’s t test. The criterion for significance was set at p<0.01.

### Statistical Analysis

Statistical analysis of the data was performed using using the SPSS statistical software for Windows (version 15 and 19; SPSS Inc., Chicago, IL). The data are compositional, since they convey relative rather than absolute quantities. Therefore, we transform the data using the logit transformation. Thus, each variable is of the form log(peak/(1-peak)).

Repeated measures analysis was used to determine the change in glycosylation over time and between groups. A repeated measures model was fitted for the logit transform of each glycan peak. The Greenhouse Geisser adjustment was used to account for non-homogeneity of variance across groups. The p-values were corrected for multiple testing error, using the false discovery rate (FDR) approach proposed by Benjamini and Hochberg [26]. An adjusted p<0.05 was considered statistically significant.

Pearson’s correlation was applied for correlation analyses (normal distribution).

## Results

### Tumour model

OVCAR3 cells in PBS/Matrigel were injected intraperitoneally in female C.B-17/lcr (scid/scid) mice of 8–12 weeks on day 0. We investigated influences of tumour progression and drug treatments on the serum glycome in six randomized cohorts: group 1 mice treated sc with ASA; group 2 mice treated with PBS (control for group 1, PBS sc); group 3 mice treated ip with TG; group 4 mice treated with PBS (control for groups 3 and 5, PBS ip); group 5 mice treated ip with COAM; group 6 mice not treated, only intraperitoneal injection of OVCAR3 tumor cells and group 7 consisted of mice with no tumour (blank). The blood samples were taken from all mice every week for seven weeks. ASA was administered daily by subcutaneous injection, whereas TG was administered intraperitoneally twice per week and COAM once per week. Therefore, two control groups were established which were treated with PBS in exactly the same way as the COAM and ASA groups.

### Tumour volume and immunological profiles

**Figure 1:**
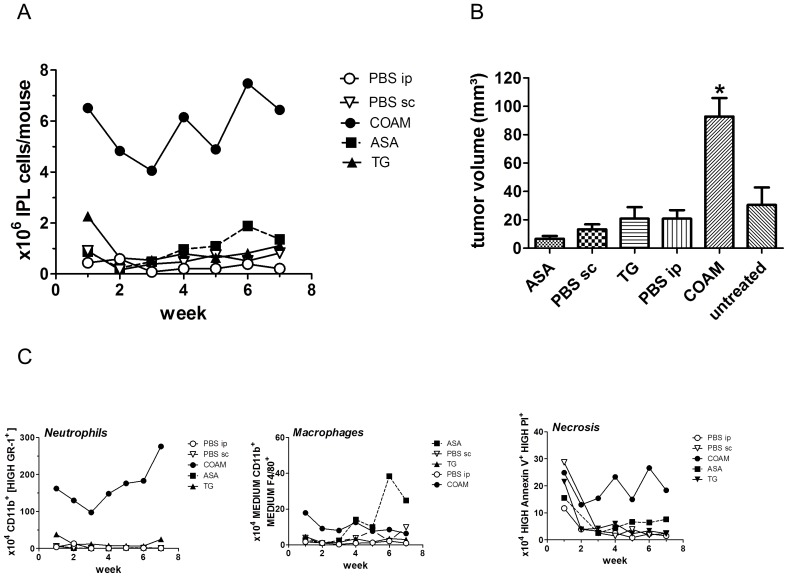
COAM-treated mice have significantly higher cell numbers, tumour volumes and numbers of neutrophils, macrophages and necrosis. **A** Total numbers of recovered viable intraperitoneal leukocytes per pool in each group (treated with COAM, ASA, TG, PBS (i.p. or s.c.); Cell counts determined in Bürker chambers (in the presence of trypan blue exclusion of dead cells), represented as mean counts per mouse. **B** Tumour volume per mouse at 7 weeks after tumour cell inoculation; *p<0.05. **C** Flow cytometry data represent absolute cell counts of pooled peritoneal cells per group (indicated as mean number per mouse, 5 mice per group): CD11B+/Gr-1+ neutrophils, CD11B+/F4/80+ macrophages and necrotic cells, as determined by propidium iodide and annexinV analysis.

Total numbers of recovered viable intraperitoneal leukocytes per group were counted weekly for each treatment group and at the end of the seventh week, tumour volumes were measured in all groups (Figure 1A–B). COAM-treated mice showed significantly higher cell numbers and tumour volumes than all other experimental groups (*p<0.05, Figure 1A–B).

The panels in Figure 1C show peritoneal cell populations. In line with previous data about cellular effects of COAM after intraperitoneal injection [14], in the COAM-treated group, significantly higher numbers of neutrophils (CD11b+ and high levels of GR-1 membrane staining) were consistently observed over the 7 week period of the experiment. Macrophage numbers were also consistently increased by COAM and also gradually increased from the third week onwards by treatment with ASA. Cell necrosis remained at high levels in the COAM groups, whereas it gradually decreased to basis levels in the other treatment groups (Figure 1C).

### Mouse serum *N*-glycome characterization

Total serum *N*-glycans from all mice samples were analysed by HILIC- and WAX-HPLC, combined with exoglycosidase digestions with structural assignments made using database matching (GlycoBase; glycobase.nibrt.ie). Assignments were also confirmed by negative ion electrospray MS [25]. *N*-glycans were separated by WAX-HPLC first and then each peak was run on HILIC-HPLC and structures were assigned with exoglycosidase digestions.

**Figure 2:**
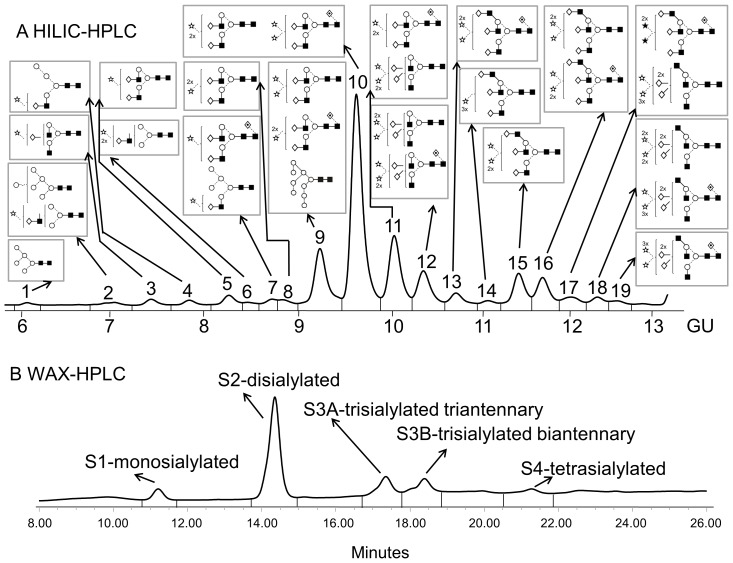
Typical HILIC- (A) and WAX-HPLC (B) chromatograms of mouse serum *N*-glycans. Structural assignments are in Table 1 and Figure S1. The HILIC-chromatogram was separated into 19 peaks and the WAX-chromatogram was separated into 5 peaks: S1, S2, S3A, S3B and S4. Symbols encode the following monosaccharide structures: GlcNAc, filled square; mannose, open circle; galactose, open diamond; fucose, diamond with a dot inside; Neu5Gc sialic acid, star with dot inside; beta linkage, solid line; alpha linkage, dotted line (Harvey et al [36]).

Serum *N*-glycans were separated into 19 peaks on HILIC-HPLC (Figure 2A) and into 5 peaks on WAX-HPLC (Figure 2B). A summary of all *N*-glycans from mouse serum is shown in Table 1 and 2 and detailed analysis in Figure S1. The whole mouse *N*-glycome contained low levels of non-sialylated glycans as well as monosialylated, disialylated biantennary, trisialylated triantennary and biantennary, and tetrasialylated triantennary glycans (Table 1). Triantennary glycans were branched at the 6-antenna (β1,6-branching, Figure S2).

**Table 1:** Summary of mouse serum *N*-glycome.

Peak	GU	ESI^1^ *m/z*	Abbreviation^2^
1	6.16	1331.6^a^	M5
2	6.99	1493.5^a^	M6
		1581.5^b^	A1G(4)1S(6)1
3	7.39	1784.6^b^	A2G(4)1S(6)1
4	7.80	1743.6^b^	M4A1G(4)1S(6)1
5	8.24	1946.7^b^	A2G(4)2S(6)1
6	8.47	–	A1G(4)1S(6,6)2
7	8.76	1905.6^b^	M5A1G(4)1S(6)1
		2092.7^b^	FA2G(4)2S(6)1
8	8.88	2253.8^b^,2275.7b,c,1126.4^d^,1186.3c,e	A2G(4)2S(3)2
9	9.34	2253.8^b^,2275.7b,c,1126.4^d^,1186.3c,e	A2G(4)2S(3,6)2
		2399.8^b^,2421.8b,c,1199.4^d^,1259.4c,e	FA2G(4)2S(3)2
		1979.6^a^	M9
10	9.78	2253.8^b^,2275.7b,c,1126.4^d^,1186.3c,e	A2G(4)2S(6)2
		1199.4^d^,1259.4c,e	FA2G(4)2S(3,6)2
11	10.21	1199.4^d^	FA2G(4)2S(6)2
		1279.9^d^,1290.9c,e	A2G(3,4)2S(3,3,6)3
12	10.53	1279.9^d^,1290.9^d^	A2G(3,4)2S(3,6,6)3
		1352.9^d^,1363.9c,e	FA2G(3,4)2S(3,3,6)3
13	10.85	1352.9^d^,1363.9c,e	FA2G(3,4)2S(3,6,6)3
14	11.16	1462.5^d^,1473.5c,e	A3G(4)3S(3)3
15	11.41	1462.5^d^,1473.5c,e	A3G(4)3S(3,3,6)3
16	11.63	1462.5^d^,1473.5c,e	A3G(4)3S(3,6,6)3
		1535.5^d^,1546.5c,e	FA3G(4)3S(3,3,6)3
17	11.92	1535.5^d^,1546.5c,e	FA3G(4)3S(3,6,6)3
		1616.0^d^,1627.0c,e,1638.1e,f	A3G(3,4,4)3S(3,3,3,6)4
18	12.26	1627.0^d^,1638.0c,e	A3G(3,4,4)3S(3,3,6,6)4
		1689.1^d^,1700.2c,e	FA3G(3,4,4)3S(3,3,3,6)4
19	12.54	1689.1^d^,1700.2c,e	FA3G(3,4,4)3S(3,6,6,6)4
S1	–		monosialylated
S2	–		disialylated biantennary
S3A	–		trisialylated triantennary
S3B	–		trisialylated biantennary
S4	–		tetrasialylated triantennary

1 Various ions:

a Singly charged ion ([M+H_2_PO_4_]^−^),

b Singly charged ion ([M-H]^−^),

c Sodium salt,

d Doubly charged ion ([M-2H]^2−^),

e Doubly charged ion ([M-H+H_2_PO_4_]^2−^),

f Di-sodium salt.

2 All *N*-glycans have two core GlcNAcs; Mx = high-mannose where x = the number of mannose residues, D = substitution position of high-mannose glycans, Ax = number of antennae, Gx = number of galactose residues, Sx = number of sialic acids, F = core fucose. The linkage positions of the galactose and sialic acid residues are given in parentheses (Royle *et al* [19]).

**Table 2:** Masses and structures of the *N*-glycans after desialylation with acetic acid.

m/z		Abbreviation^1^
MALDI ([M+Na]^+^)	ESI ([M+H_2_PO_4_]^−^)	
1501.7	1575.5	A2G(4)1
1666.6	1737.6	A2G(4,4)2
1809.6	1883.7	FA2G(4)2
2028.7	2102.7	A3G(4)3

All peaks in all samples were plotted to monitor whether a trend of different progression or significant differences existed among groups *vide infra*.

### Increased sialylation and branching indicates the presence of ovarian tumours in mice and pro-inflammatory drugs significantly alter these glycosylation changes

Serum *N*-glycomes from untreated mice carrying tumour and mice without tumour (blank) were compared. HILIC-HPLC profiles show decreases in high mannosylated and monosialylated glycans that indicates tumour presence (Table 3).

**Table 3:** Average % glycan values of significantly altered peaks between untreated tumour-bearing mice versus mice without tumours (blank).

HILIC				
Peak2		Peaks		
M6+A1G(4)1S(6)1		Abbreviation		
Group 7	Group 6	Group		
1.43	1.60	Week 0	Average values of % peak areas in group	**A** Untreated tumour-bearing mice versus mice without tumours (blank)
1.10	1.09	Week 1		
1.50	1.18	Week 2		
1.59	1.53	Week 3		
1.14	1.24	Week 4		
1.15	1.10	Week 5		
1.44	1.25	Week 6		
1.24	1.131	Week 7		
0.889		Group	FDR adjusted P-value	
**↓0.020**		Progression		

Significant p-values are highlighted in bold (p<0.05) (peak 2 is significantly decreased).

*N*-glycomes from ASA-, TG- [16] and COAM-treated [14], [15] mice were compared to control mice.

No significant differences were observed after ASA-treatment. In TG- and COAM-treated mice, both HILIC- and WAX-HPLC showed an increase in highly sialylated and branched glycans and a decrease in less branched and sialylated glycans, indicating an increase in branching and sialylation with TG- and COAM-treatment (Table 4 and 5). These changes were more significant with COAM-administration, where almost the complete *N*-glycome was affected (Table 5).

**Table 4:** Average % glycan values of significantly altered peaks between TG-treated versus control (PBS ip).

WAX - HPLC				HILIC						
Peak S4		Peak S1		Peak 14		Peak 12		Peaks		
Tetrasialylated triantennary		Monosialylated		A3G(4)S(3)3		A2G [3], [4]S(3,6,6)3+FA2G [3], [4]S(3,3,6)3		Abbreviation		
Group 4	Group 3	Group 4	Group 3	Group 4	Group 3	Group 4	Group 3	Group		
2.69	3.19	6.46	6.98	0.59	0.85	5.39	5.72	Week 0	Average values of % peak areas in group	**B** TG-treted versus control (PBS ip)
3.18	3.32	6.37	6.87	0.94	1.11	7.32	7.95	Week 1		
3.10	3.71	6.54	6.04	0.85	0.98	7.02	7.81	Week 2		
2.90	4.12	7.36	6.89	0.68	0.83	5.15	6.68	Week 3		
3.16	3.82	7.24	6.44	0.59	0.93	5.90	7.10	Week 4		
3.20	3.90	7.16	6.88	0.68	0.93	6.08	7.30	Week 5		
2.72	3.70	7.16	6.88	0.84	1.22	6.32	7.68	Week 6		
2.84	3.72	7.64	6.93	0.77	0.89	6.01	6.44	Week 7		
**↑0.005**		**↓0.038**		**↑0.050**		**↑0.050**		Group	FDR adjusted P-value	
0.263		**↓0.040**		0.746		0.661		Progression		

Significant p-values are highlighted in bold (p<0.05) (peaks 12, 14 and S4 are significantly increased and peak S1 is significantly decreased).

**Table 5:** Average % glycan values of significantly altered peaks between COAM-treated versus control (PBS ip).

WAX - HPLC						HILIC																																
Peak S4		Peak S3B		Peak S2		Paek 18		Peak 17		Peak 15		Peak 14		Peak 12		Peak 11		Peak 10		Peak 9		Peak 8		Paek 7		Paek 6		Peak 5		Peak 4		Peak 3		Peak 2		Peaks		
Tetrasialylated triantennary		Trisialylated biantennary		Disialylated biantennary		A3G3S(3,3,6,6)4+FA3G3S(3,3,3,6)4		FA3G3S(3,6,6)3+A3G3S(3,3,3,6)4		A3G3S(3,3,6)3		A3G(4)S(3)3		A2G [3], [4]S(3,6,6)3+FA2G [3], [4]S(3,3,6)3		FA2G2S(6)2+A2G(3,4)2S(3,3,6)3		A2G2S(6)2+FA2G2S(3,6)2		A2G2S(3,6)2+FA2G2S(3)2		A2G2S(3)2		M5A1G1S(6)1+FA2G2S(6)1		A1G1S(6)2		A2G2S(6)1		M4A1G1S(6)1		A2G1S(6)1		M6, A1G1S(6)1		Abbreviation		**C** COAM-treated versus control (PBS ip)
Group 5	Group 4	Group 5	Group 4	Group 5	Group 4	Group 5	Group 4	Group 5	Group 4	Group 5	Group 4	Group 5	Group 4	Group 5	Group 4	Group 5	Group 4	Group 5	Group 4	Group 5	Group 4	Group 5	Group 4	Group 5	Group 4	Group 5	Group 4	Group 5	Group 4	Group 5	Group 4	Group 5	Group 4	Group 5	Group 4	Group		
2.83	2.69	12.89	13.38	63.61	63.90	0.74	0.76	1.81	1.89	2.15	2.14	0.62	0.59	5.26	5.39	10.29	10.38	42.52	43.57	13.94	13.79	1.58	1.34	2.30	2.40	1.12	1.01	5.67	5.20	2.36	2.20	2.15	2.06	1.95	1.78	Week 0	Average values of % peak areas in group	
5.38	3.18	21.27	12.65	56.17	65.35	1.52	1.13	2.89	3.63	4.59	4.26	2.65	0.94	13.33	7.32	9.75	12.01	38.34	42.31	11.53	12.44	1.08	1.26	1.74	1.52	0.64	0.77	2.79	3.21	1.28	1.36	0.91	1.35	1.06	1.11	Week 1		
5.73	3.10	19.21	12.64	57.53	65.64	2.49	0.78	4.15	2.92	5.82	3.51	2.68	0.85	12.98	7.02	10.23	11.74	37.33	43.24	10.04	12.81	0.78	1.34	1.34	1.77	0.52	0.89	1.96	3.70	0.96	1.57	0.73	1.47	0.85	1.31	Week 2		
5.04	2.90	19.03	12.50	58.71	64.58	1.44	0.84	2.33	2.07	3.41	2.43	1.97	0.68	10.53	5.15	9.99	10.21	38.63	40.57	13.00	13.63	1.53	1.55	2.04	2.58	0.78	1.00	3.89	5.55	1.72	2.38	1.22	2.15	1.37	2.08	Week 3		
5.54	3.16	17.15	11.02	58.52	66.48	1.43	0.54	3.02	2.22	4.34	2.72	1.90	0.59	10.87	5.90	10.60	11.84	39.15	43.41	11.50	13.51	1.13	1.45	2.03	2.23	0.82	1.02	3.44	4.59	1.52	1.99	1.12	1.80	1.26	1.68	Week 4		
5.82	3.20	17.83	10.00	57.31	66.74	1.64	0.65	3.41	2.76	5.37	3.37	2.41	0.68	12.12	6.08	9.48	11.57	38.08	43.23	12.02	13.50	1.11	1.38	1.77	2.01	0.55	0.85	2.81	4.17	1.31	1.83	1.03	1.74	1.10	1.51	Week 5		
6.59	2.72	19.18	11.27	54.84	67.53	1.83	0.96	3.58	3.28	5.58	3.66	2.63	0.84	12.67	6.32	9.94	13.14	36.76	41.44	10.97	12.12	0.94	1.12	1.79	1.92	0.60	0.76	2.64	3.80	1.26	1.54	0.97	1.48	1.22	1.38	Week 6		
4.97	2.84	13.25	9.33	61.70	68.89	1.77	1.33	4.49	4.67	5.90	4.71	1.40	0.77	8.63	6.01	11.73	13.88	37.48	40.68	11.55	11.25	0.96	1.04	1.74	1.60	0.60	0.80	2.94	3.00	1.43	1.32	1.24	1.41	1.35	1.21	Week 7		
**↑0.002**		**↑0.002**		**↓<0.01**		**↑0.007**		**↑0.011**		**↑0.011**		**↑0.000**		**↑0.000**		**↑0.010**		**↓0.001**		**↓0.041**		**↓0.011**		**↓0.039**		**↓0.009**		**↓0.004**		**↓0.017**		**↓0.002**		**↓0.023**		Group	FDR adjusted P-value	
0.354		0.220		**↓0.015**		0.163		0.207		0.301		**↑0.027**		**↑0.027**		0.176		0.271		**↓0.050**		**↓0.027**		0.301		0.174		0.163		0.174		0.163		0.174		Progression		

Significant p-values are highlighted in bold (p<0.05) (peaks 2, 3, 4, 5, 6, 7, 8, 9, 10 and S2 are significantly decreased and peaks 11, 12, 14, 15, 17, 18, S3B and S4 are significantly increased).

### Increases in sialylation in serum glycoproteins are associated with tumour progression

**Figure 3:**
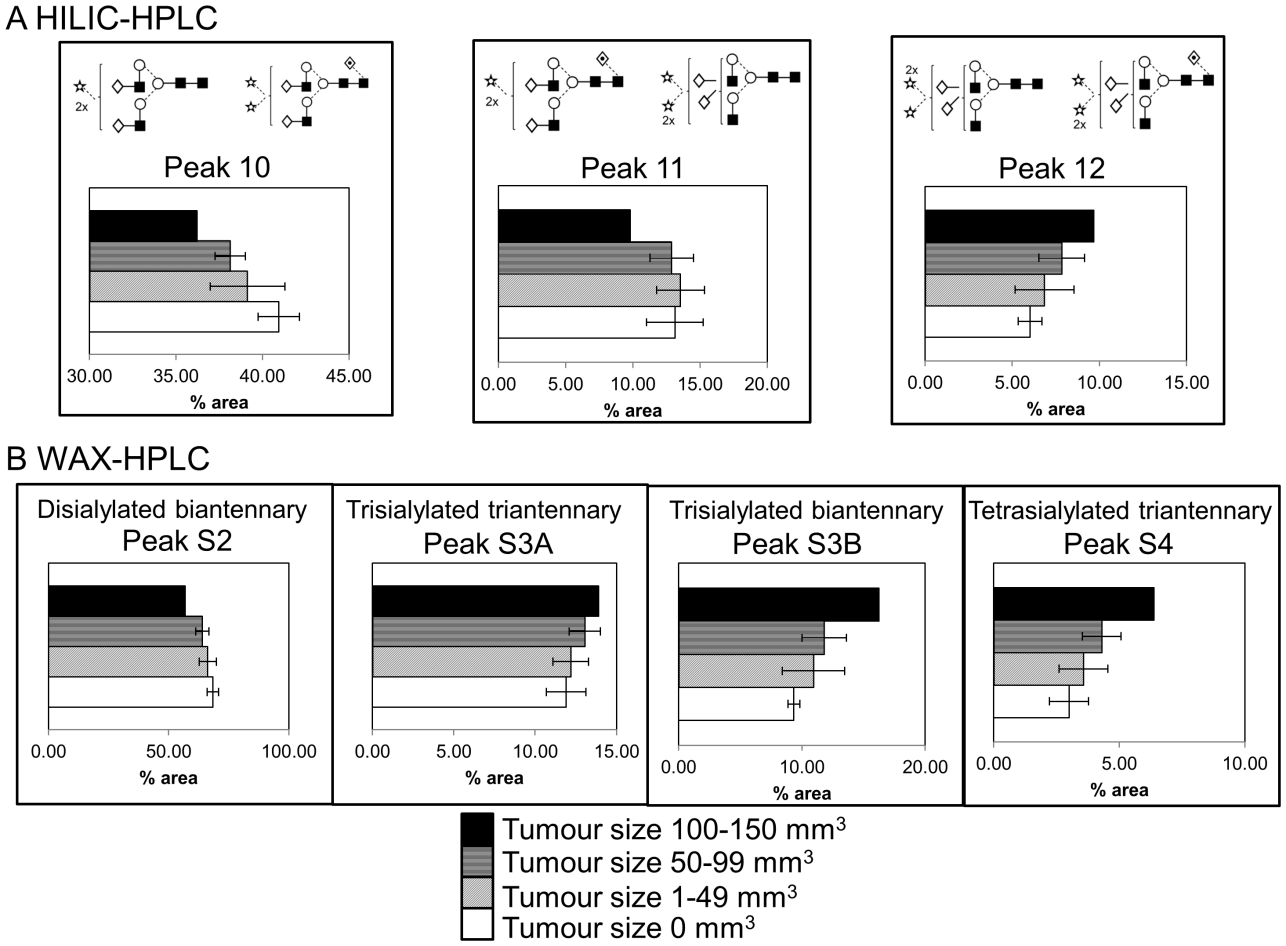
Serum glycoprotein sialylation significantly increases with tumour volume. Significant correlations of tumour volume with HILIC- (**A**) and WAX- (**B**)-peaks. Data represent mean peak %area per mouse, 5 mice per group.

After the changes in the serum *N*-glycomes from mice in each group were identified, tumour volumes were correlated with the *N*-glycomes. Both HILIC- and WAX-HPLC showed a decrease in disialylated and an increase in trisialylated glycans and WAX-HPLC also showed an increase in tetrasialylated glycans that correlated with increased tumour size (Figure 3, Table S1).

We have further looked into sialylation in these samples and also expression of enzymes responsible for branching and sialylation on mouse glycans.

### Sialic acid analysis shows high proportions of glycolylneuraminic acid and no observed changes among the treatment groups

Sialic acids from mice sera were examined. Samples contained mostly *N*-glycolylneuraminic acid (Figure S3). There were no observed correlations in the proportions of the sialic acid species with treatments or tumour volumes. Murine HILIC peaks eluted at higher GUs than human glycans because of the presence of Neu5Gc instead of Neu5Ac and probably also due to the linkages of some sialic acids to GlcNAc [27], [28], [29]. Also sialidase digestions on some samples required higher amount of the enzyme suggesting a less accessible linkage of sialic acid on GlcNAc compared with Gal.

### Expression of glycosyltransferases in mouse liver shows consistence in increasing sialylation and branching with tumour and pro-inflammatory COAM administration

The expression of several glycosyltransferases related to branching and sialylation was analysed in the livers from treated and untreated mice and mice without tumour (blank).

**Figure 4:**
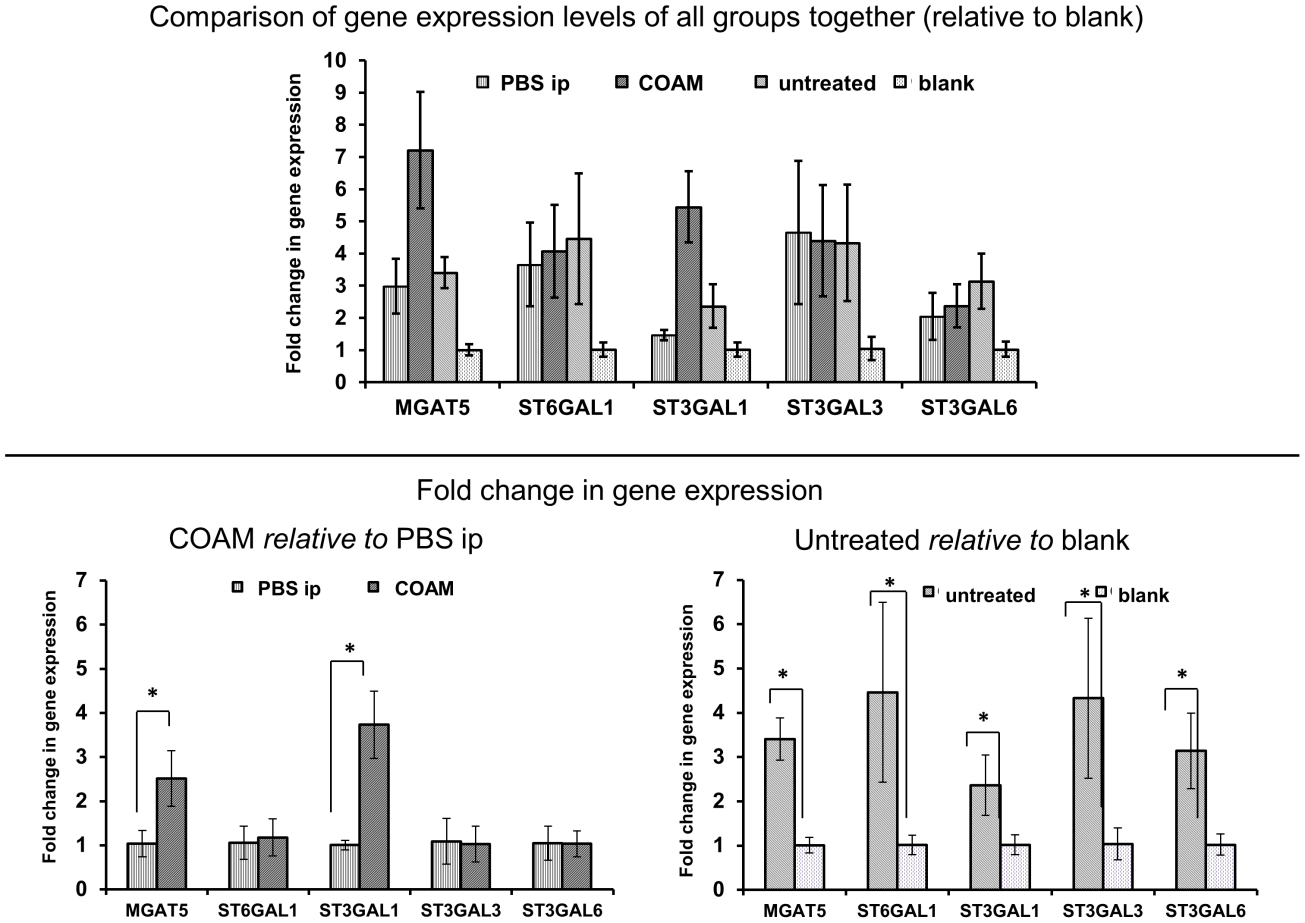
Sialyltransferases and branching enzyme levels significantly increase in tumour-bearing mice and with COAM-treatment. Plotted is the relative mRNA expression of glycosyltransferase enzymes in mouse liver in four mouse groups (Untreated tumour-bearing mice compared to mice without the tumour (blank) and COAM-treated mice compared to control (PBS ip)). *p<0.01; Bars indicate standard deviation of four independent experiments which were comprised of four replicates each.

The presence of the tumour is associated with up-regulated α1,6-mannosylglycoprotein β1,6-*N*-acetylglucosaminyltransferase5 (MGAT5) by 3.4-fold and several α2,3-siayltransferases that transfer α2,3-sialic acid on terminal galactose, ST3Gal1 (2,3-fold), ST3Gal3 (4.2-fold) and ST3Gal6 (3.1-fold), as well as an increase in the expression of α2,6-sialtransferase1 (ST6Gal1) by 4.4-fold (Figure 4). ST3Gal1 transfers α2,3-sialic acid onto Gal-β1-3GalNAc on *O*-glycans and glycolipids, ST3Gal3 acts on Gal-β1-3(4)GlcNAc, ST3Gal6 acts on Galβ1-4GlcNAc and ST6Gal1 acts on Gal-β1-4GlcNAc on *N*- or *O*-glycans. As a result, an increase in branching and sialylation on the liver glycoproteins from tumour-bearing mice was expected on the basis of these expression changes. Indeed, the overexpression of those sialyltransferases was consistent with the increase in sialylation in tumour-bearing mice.

Livers from COAM-treated mice showed an overexpression of MGAT5 by 2.4-fold and ST3Gal1 in 3.7-fold compared to controls (Figure 4). This is consistent with the increase in branching on *N*-glycans from these mice. The increase in sialylation could be explained by the up-regulation of MGAT5, which gives rise to β1,6-branching, which was found present on triantennary glycans (Figure S2). This new branch could be extended with terminal galactose residues that may be sialylated and thus lead to an increase in tri- and tetrasialylated glycans.

The expression of all sialyltransferases and MGAT5 was significantly increased in the tumour bearing mice compared to mice without tumours (blank) (Figure 4).

## Discussion

In a previous pre-clinical ovarian carcinoma study, related to inflammation and cancer, TG was used as an inflammatory stimulus to elicit peritoneal macrophages [16]. We here used the same animal model and compared COAM with TG and observed that (i) COAM is a more potent pro-inflammatory activator of peritoneal myeloid cells and (ii) its effects on glycosylation of serum proteins was more extended than those observed with TG. In addition, we provide a means of monitoring tumour progression by measuring serum glycome markers. We also provide a general approach monitoring the effect of tumour-associated inflammation on disease progression and serum marker alterations. COAM induces and binds chemokines in such a way that these molecules remain chemotactically active [14], [15]. The bound chemokines, for instance GCP-2/CXCL6, exert a potent neutrophil chemotaxis in the mouse [30]. Myeloid cells, depending on their polarization, have dual effects on cancer progression [31]. In this study, we observed by FACS analysis the presence of myeloid cells, both neutrophils and macrophages (Figure 1C). The induction of both types of myeloid cells by COAM was corroborated in the peritoneal cavity, where the ovarian cancer progressed. Increased neutrophil-mediated inflammation, induced by COAM, which correlated with increased tumour volume (Figure 1b) adds essential complementary information to previous findings that anti-inflammatory drugs reduce the risk of cancer [7], [10]. The tumour-associated neutrophils, depending on their stimulation state and micro-environment, may have pro- or anti-tumoural effects [31]. Recently, we detected an early anti-tumoural effect of COAM and neutrophils in a syngenic melanoma mouse model. As the melanoma model progressed, the effect of COAM at later tumour developmental stages was not beneficial [15]. Together with the present data, this seems to indicate that prolongation of neutrophil influx into tumors, such as was observed in tumours with higher neutrophil chemokine levels, helps in tumour progression and is detrimental for the host [31].

### Sialylation and branching increases with administration of pro-inflammatory drugs and with tumour progression

We observed significant increases in sialylation and branching on glycans in mice treated with pro-inflammatory compounds, such as COAM and TG (Table 4 and 5). These changes in glycosylation were more significantly pronounced in case of COAM administration compared to TG. Tumour volume significantly increased with COAM administration, whereas only slightly with TG treatment (Figure 1b). We hypothesize that this may be explained by the following sequence of events. Firstly, inflammatory agents such as cytokines from the tumour promote the expression of liver glycoproteins such as acute phase proteins with altered glycosylation [32]. Then the resulting inflammatory response promotes tumour growth, therefore the changes in glycosylation may precede the changes in tumour volumes. Also, we observed increases in sialylation that correlated with increased tumour size (Figure 3). These effects were detected in serum samples, instead of biopsies. Therefore they indicate that, in the future such analysis might constitute a patient-compliant way for monitoring ovarian cancer.

Several publications report changes in glycosylation in cancer and inflammatory conditions in mouse models. Lin *et al.* found significant increases in internal α2,6-sialylation in colon and breast tumours and substitution of *N*-glycolylneuraminic acid for *N*-acetylneuraminic acid in colonic tumour using matrix-assisted laser desorption/ionization (MALDI)-MS [28]. Lattova *et al*. described increase in high mannose glycans and hybrid glycans, core-fucosylated and disialylated core-fucosylated glycans in head and neck tumours also using MALDI-MS [33]. Yasukawa *et al.* observed increase in α2,3- and α2,6-sialylation and α2,3- and α2,6-sialyltransferases using lectins and RT-PCR in inflammation [34]. Itoh *et al.* found increases in α1,6-fucose and α2,6-fucosyltransferase in diabetic mice using HPLC of pyridineaminated glycans and RT-PCR [35].

### Glycosyltransferases in liver correspond to observed increases in sialylation and branching with tumour and pro-inflammatory drugs administration

We identified several glycosyltransferases responsible for sialylation and branching of glycans attached to serum glycoproteins that were consistently expressed in mouse liver tissue. MGAT5, responsible for branching, was significantly increased in mice with tumours compared with those without, as well as in mice treated with pro-inflammatory COAM (Figure 4). Several sialyltransferases were increased in the tumor-bearing mice too, including α2,3-sialtransferases ST3Gal1, ST3Gal3 and ST3Gal6 as well as α2,6-sialtransferase1 ST6Gal1. Increase in ST3Gal1, ST3Gal3 and ST6Gal1 in the tumour-bearing hosts is consistent with data from Yasukawa *et al.* who found these enzymes also increased in mouse liver after turpentine-induced inflammation [34].

## Conclusions

Association of cancer and inflammation has been discussed for decades. Non-steroidal anti-inflammatory drugs were found to be protective and reduce the risk of cancer, decrease cell growth, induce apoptosis and inhibit the survival of cancer cells [7], [10], [11]. Therefore, here the mechanism of anti-inflammatory drugs limiting cancer progression appears to be through modulating the inflammatory response which is tightly connected to carcinogenesis. This is the first report of a direct correlation between glycosylation changes in the cancer serum glycome and inflammation as well as with treatments altering the inflammatory process in a relevant tumour model of ovarian cancer. Also, this is the first report describing the influence of COAM on tumour-associated inflammation, tumour progression and glycosylation in cancer sera. Overall, this study provides the description of an animal model and method to begin to study the mechanisms and effects of immune system modulators on glycan and glycoprotein changes on tumor biology. We introduce the novel method of looking at potential effects from tumour associated inflammatory signalling on liver released glycans and glycoproteins. The changes we have described in the serum glycome correlate with tumour progression and could serve as potentially useful serum biomarkers for monitoring cancer progression and response to therapy.

## Supporting Information

Figure S1 **Detailed** ***N*** **-glycan analysis of mouse serum on individual WAX fractions.**
(PDF)

Figure S2 **Negative ion electrospray MS/MS spectrum of the triantennary glycan (phosphate adduct) showed that triantennary glycans are branched on the 6-antenna.**
(DOC)

Figure S3 **Sialic acid speciation by DMB labelling shows mouse serum contains mostly** ***N*** **-glycolylneuraminic acid.**
(DOC)

Table S1 **Correlations of** ***N*** **-glycome and tumour volume.**
(DOC)

Table S2 **Sialyltranferases with promoters conserved among species.**
(DOC)

Methods S1 **Supplementary Materials and Methods, Results, and Discussion.**
(DOC)
