# Supplementary material for: Increase in Sialylation and Branching in the Mouse Serum N-glycome Correlates with Inflammation and Ovarian Tumour Progression
Source: PLoS One. 2013 Aug 30;8(8):e71159. doi: 10.1371/journal.pone.0071159 (PMC3758313; doi:10.1371/journal.pone.0071159)

Figure S1: **Detailed *N*-glycan analysis of mouse serum of individual WAX fractions.**

The whole mouse *N*-glycome contained monosialylated, disialylated biantennary, trisialylated triantennary and biantennary, and tetrasialylated triantennary glycans. Galactose was linked β1-3 or β1-4 based on digestions with bovine testes β-galactosidase (BTG) and *Streptococcus pneumoniae* β-galactosidase (SPG). BTG digests non-reducing terminal galactose β1-3 and β1-4 linkages, whereas SPG digests non-reducing terminal galactose β1-4 linkages. *Arthrobacter ureafaciens* sialidase (ABS) releases α2-3,6 and 8 liked non-reducing terminal sialic acids. ABS digestions on some samples required higher amount of the enzyme suggesting less accessible linkage of sialic acid on GlcNAc. *Streptococcus pneumoniae* sialidase (NAN1) releases α1-3 linked non-reducing terminal sialic acids; almond meal α-fucosidase (AMF) releases α1-3 and 4 linked non-reducing terminal fucose residues, whereas *Xanthomonus sp.* alpha-fucosidase (XMF) removes α1,2 linked fucose and bovine kidney α-fucosidase (BKF) releases α1-2 and α1-6 fucose linked non-reducing terminal fucose residues more efficiently than α1-3 and 4 linked fucose and also digests core α1-6 fucose; coffee bean α-galactosidase (CBG) hydrolyses α1-3 and α1-4 galactose; jack bean β-*N*-acetylhexosaminidase (JBH) releases non reducing terminal β (1-2,3,4,6) linked *N*-acetylglucosamine (GlcNAc) and *N*-acetylgalactosamine (GalNAc) residues; jack bean α-mannosidase (JBM) removes mannose linked α1-2,6 >3; β-*N*-acetylglucosaminidase cloned from *Streptococcus pneumonia,* expressed in *E. coli* (GUH) digests βGlcNAc, but not a bisecting GlNAc β1-4 linked to mannose.

For structural abbreviations see legend of Figure 1.

A) HILIC-HPLC of unfractionated and WAX fractionated fractions of whole mouse serum *N*-glycome

WAX0 = neutral, S1 = monosialylated, S2 = disialylated, S3A = trisialylated triantennary, S3B = trisialylated biantennary and S4 = tetrasialylated fraction.

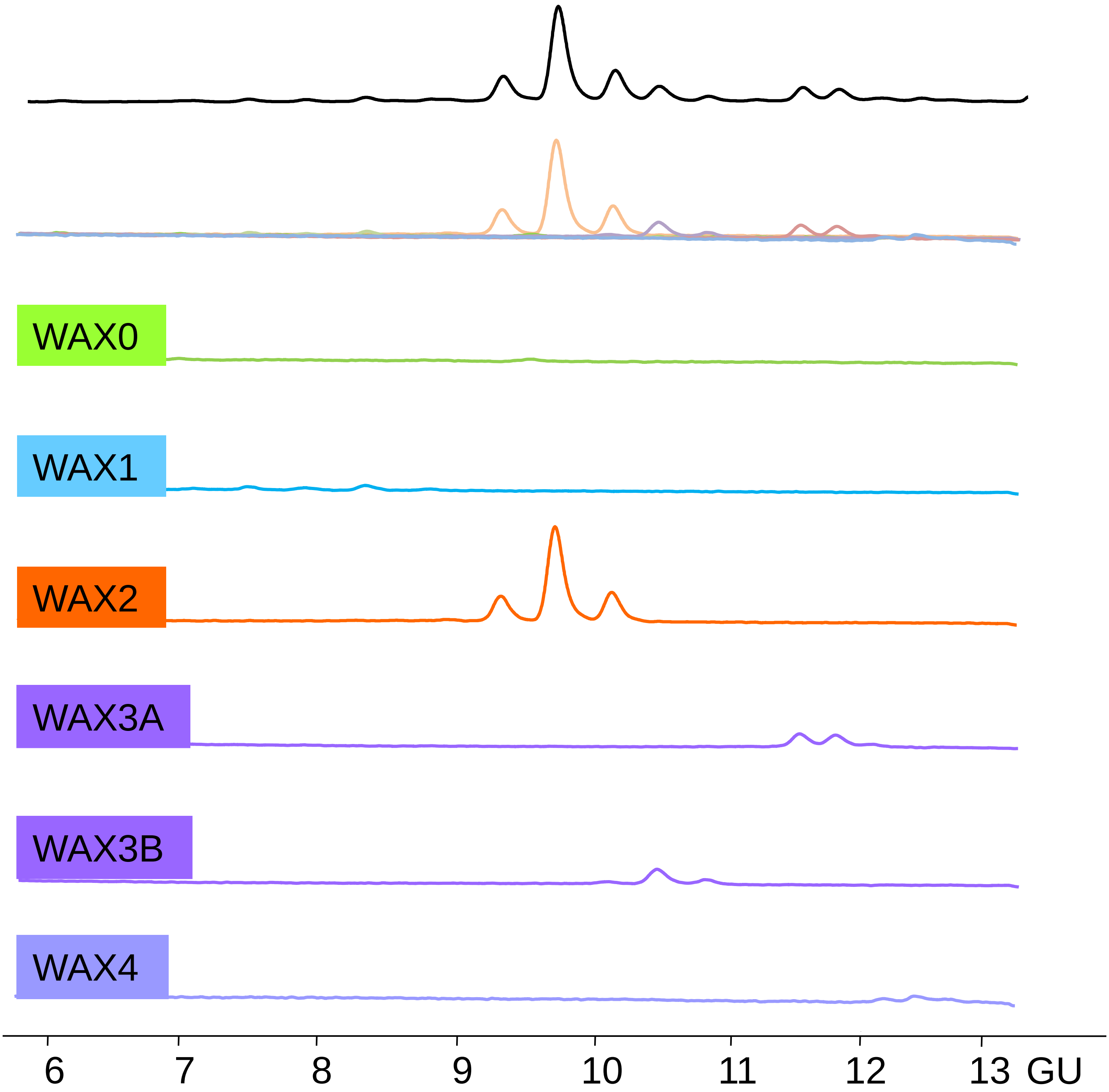

B) Exoglycosidase digests of unfractionated mouse serum *N*-glycome

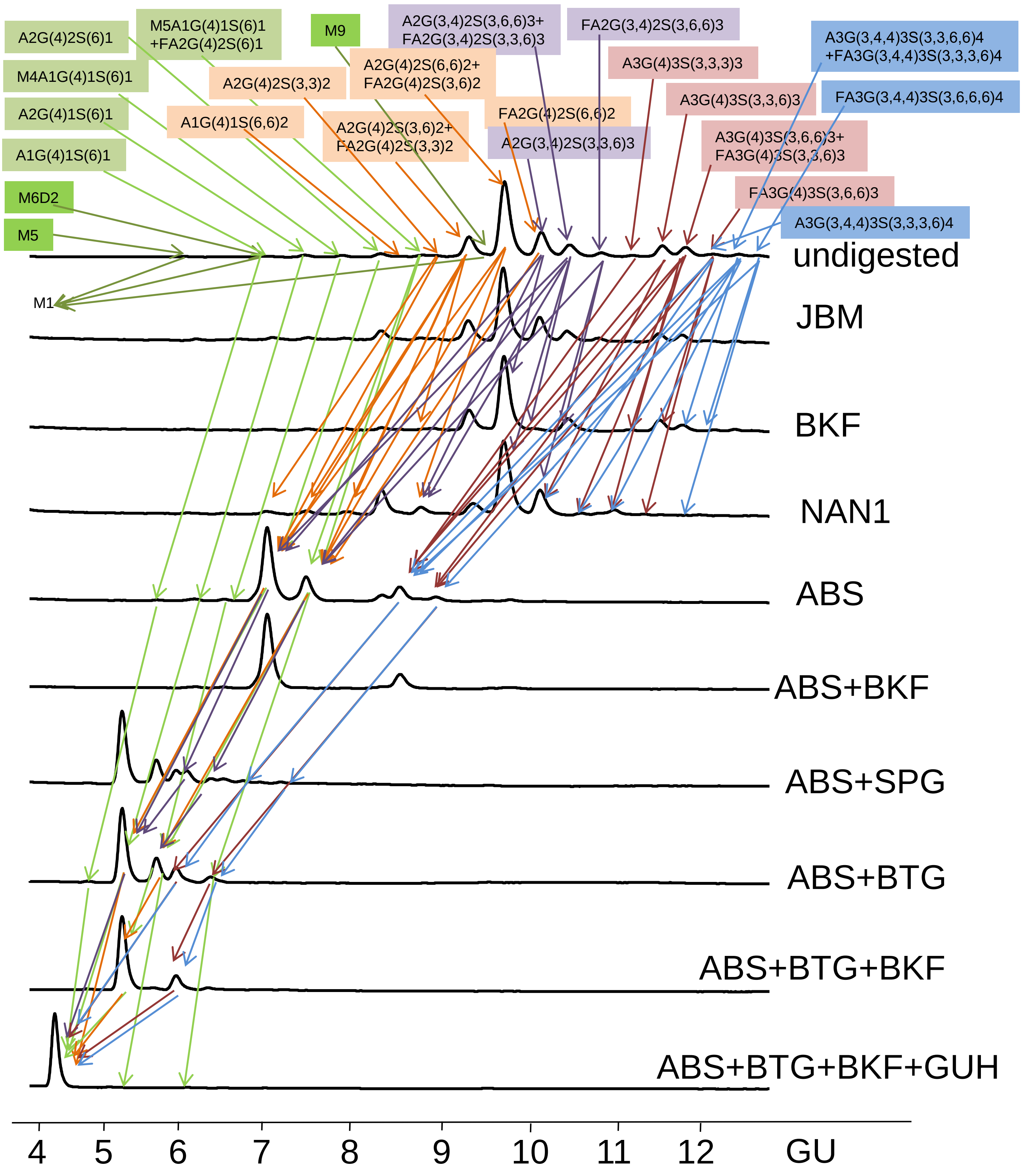

XMF, AMF, JBH and CBG did not make difference to the profiles – no outer arm fucose, no GalNAc and no alpha-galactose present in mouse serum

C) Exoglycosidase digests of WAX fractions of mouse serum *N*-glycome

Neutral (S0)

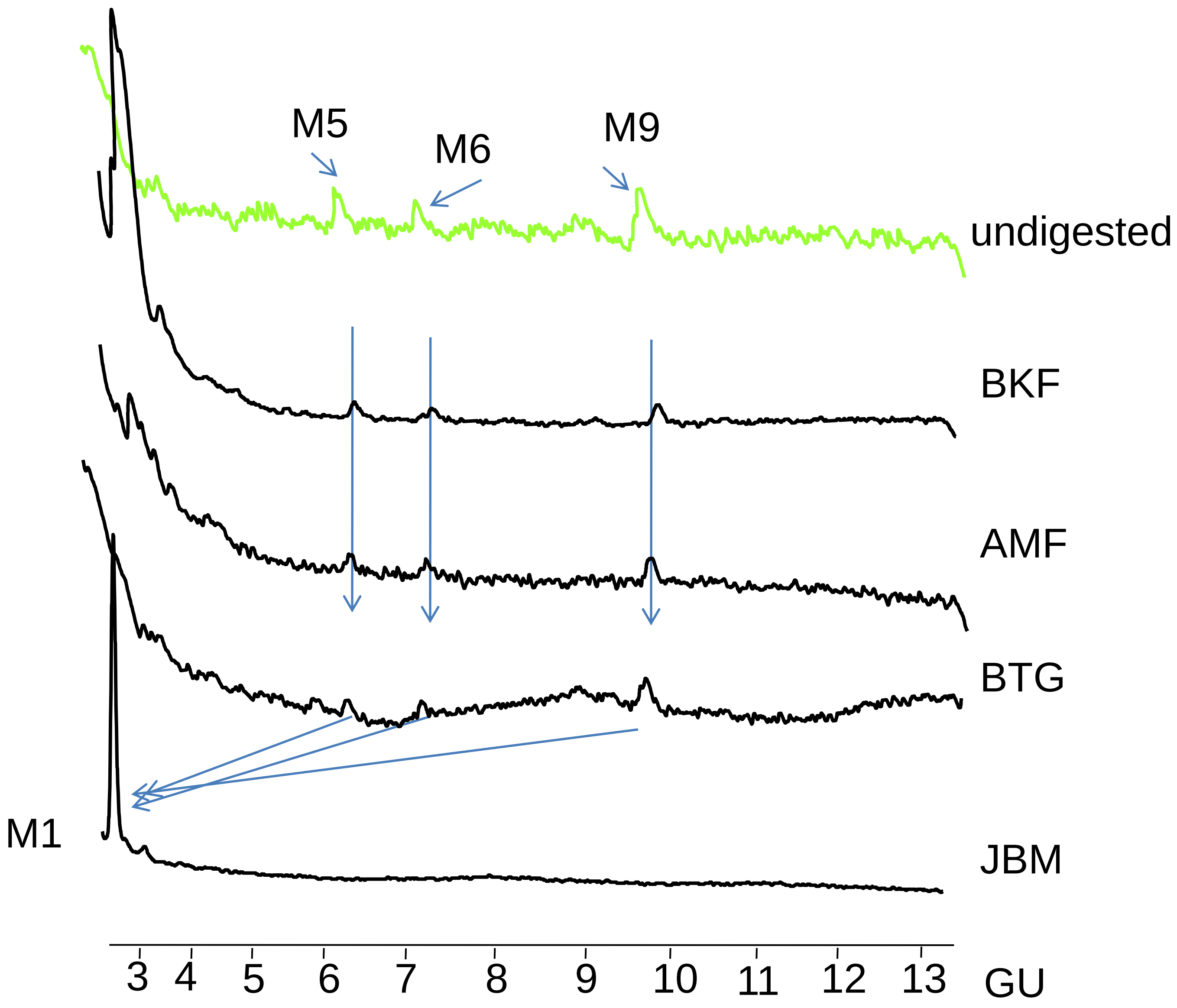

Neutral fraction contains only high mannosylated glycans

Monosialylated (S1)

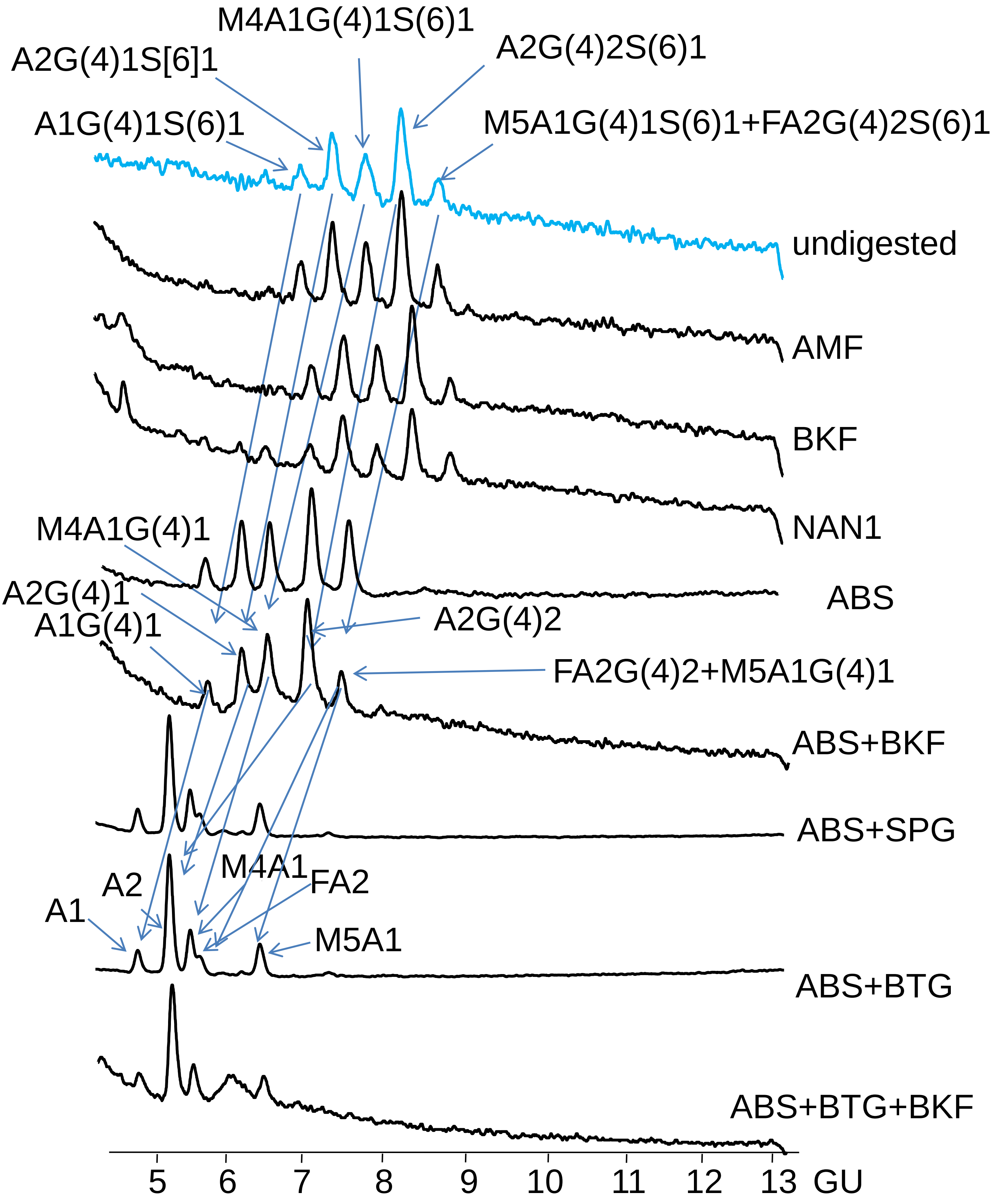

Disialylated (S2)

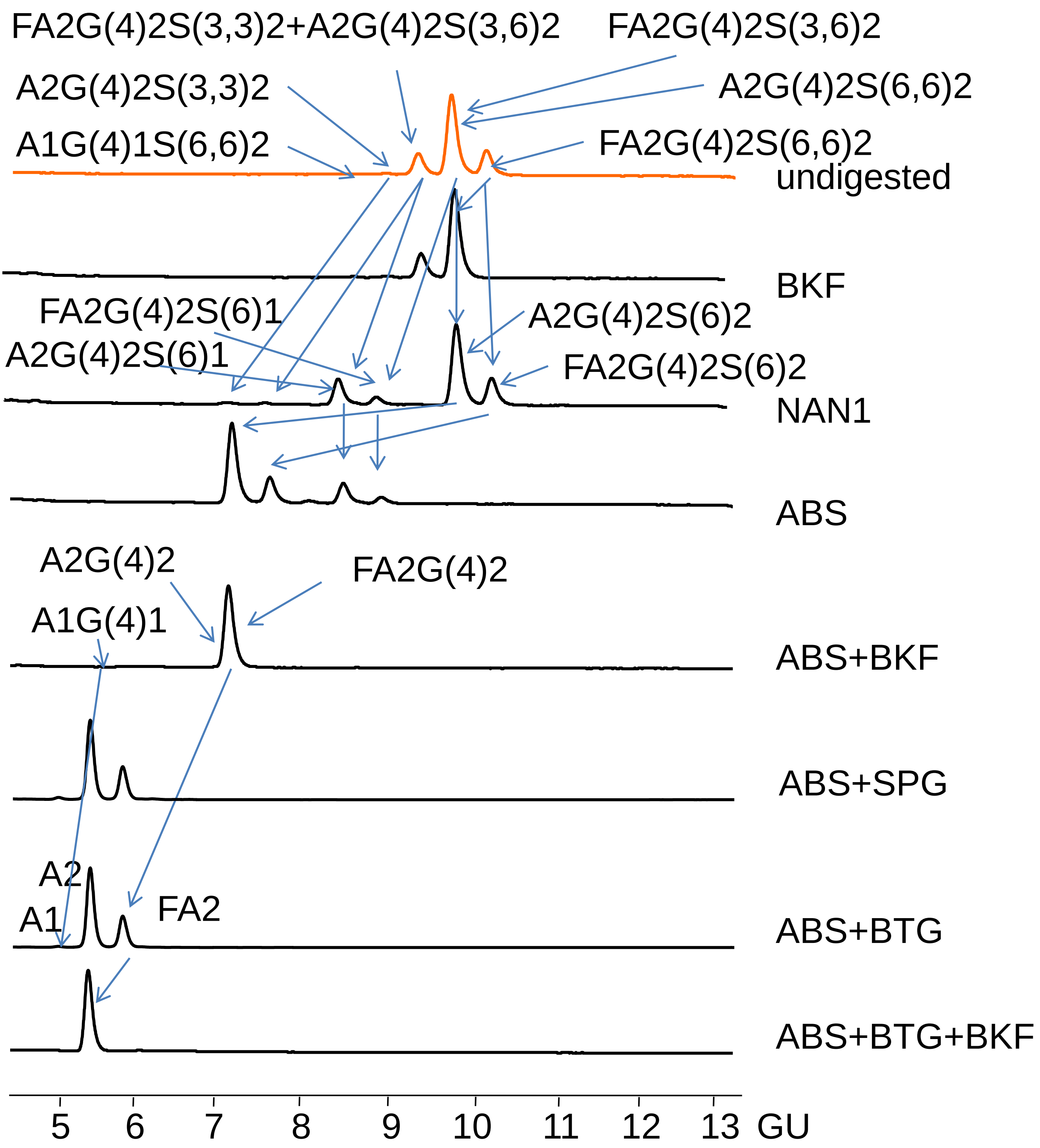

Trisialylated triantennary (S3A)

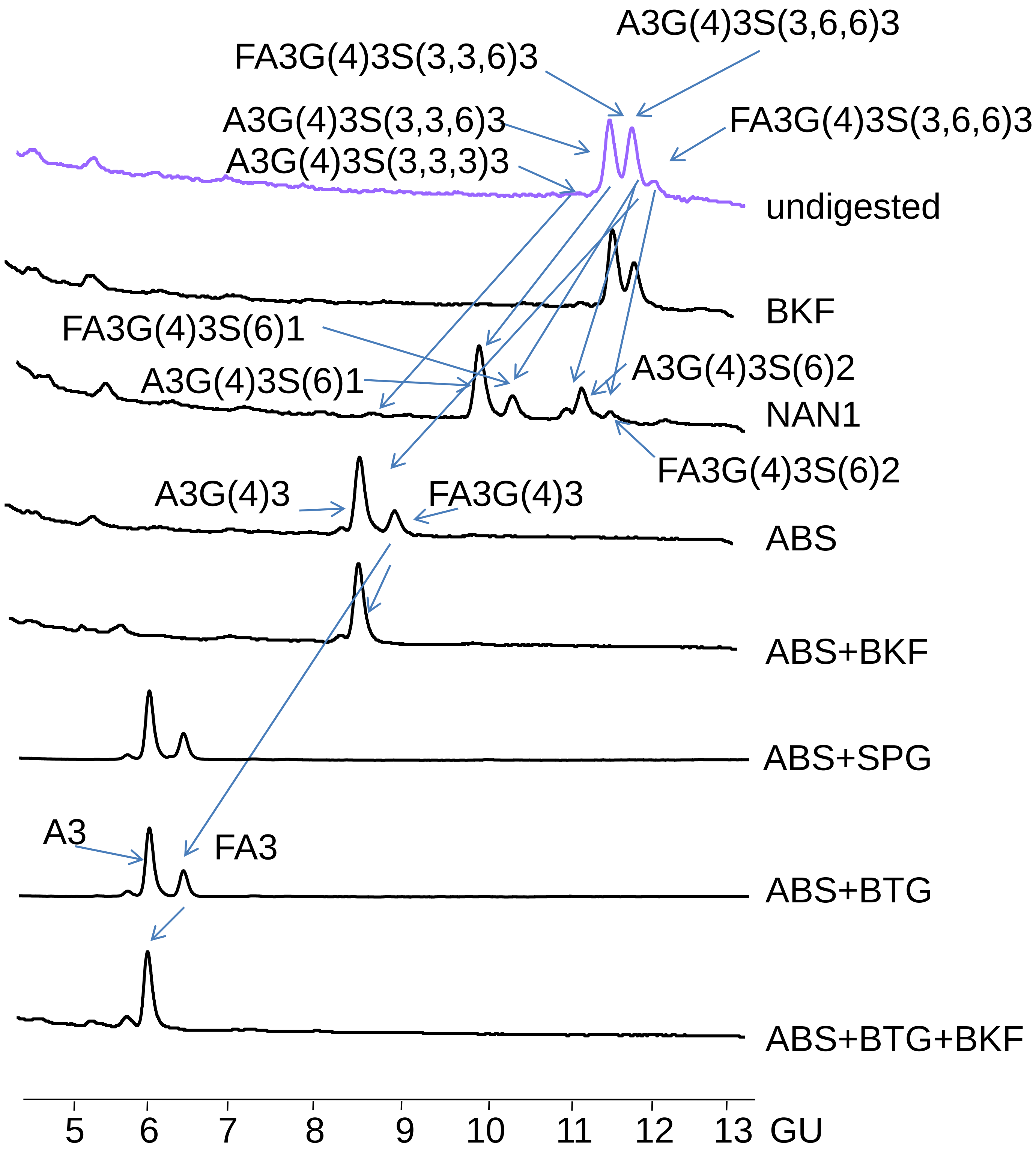

Trisialylated biantennary (S3B)

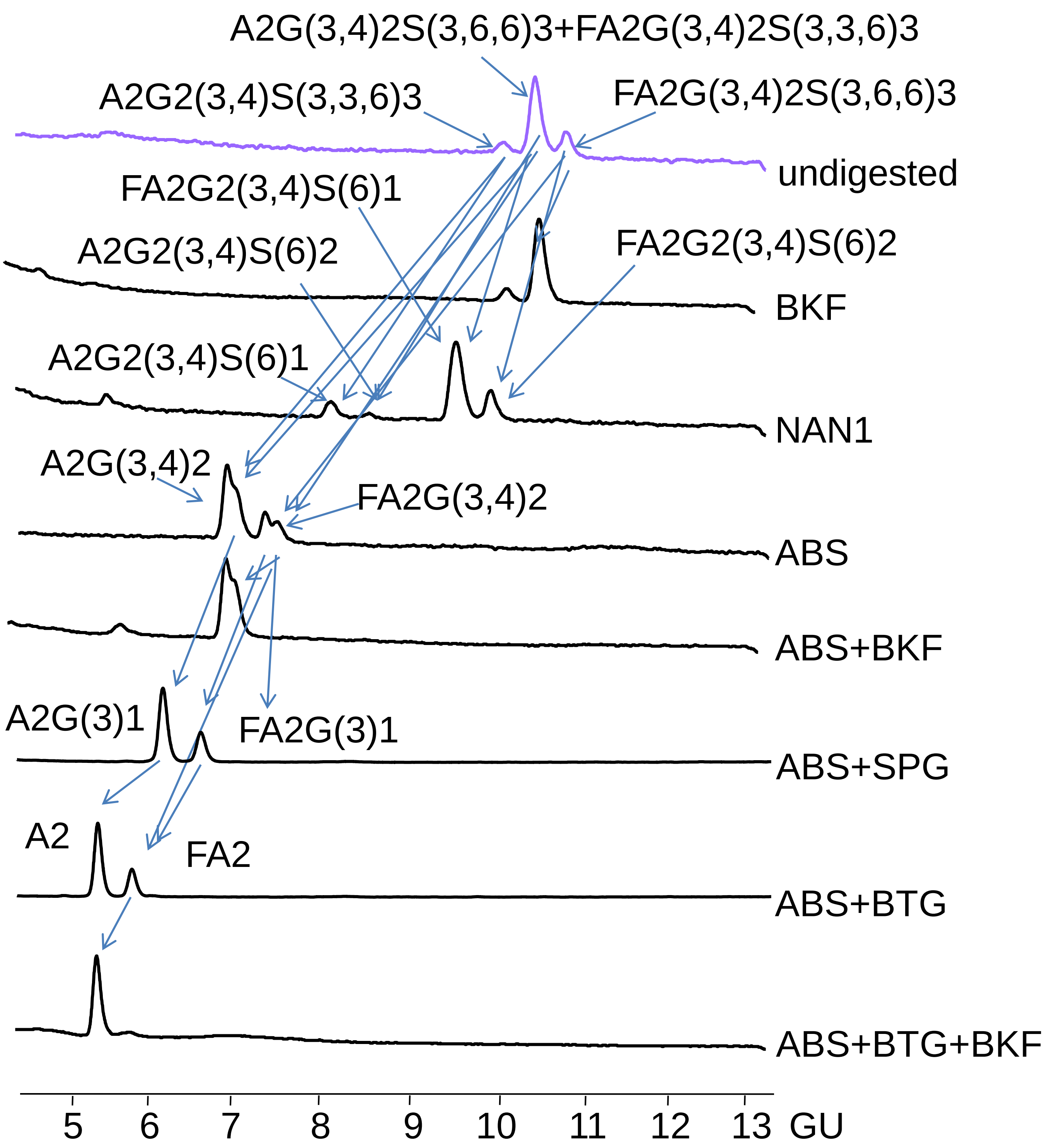

Tetrasialylated (S4)

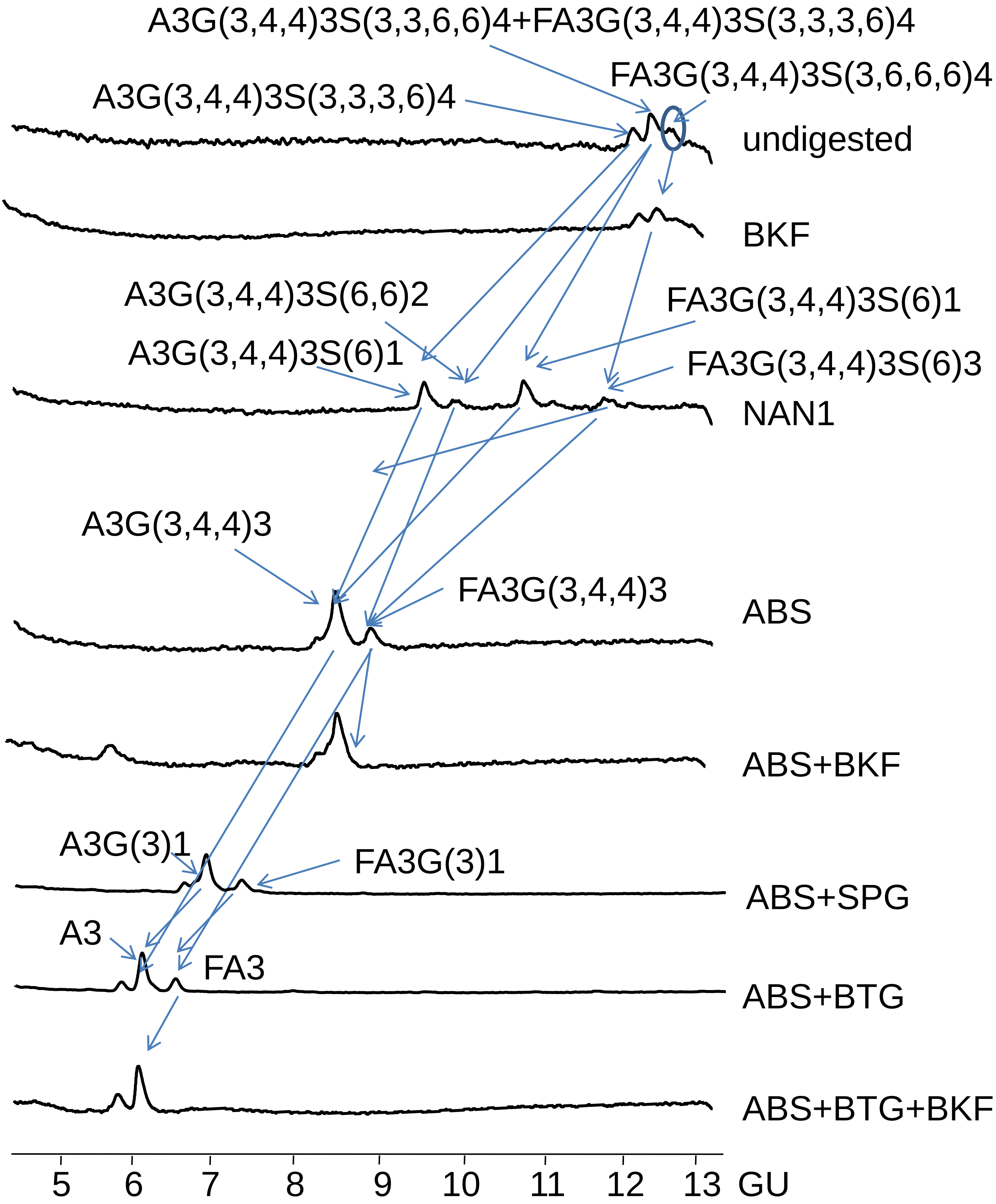

Supplement: Figure S1 — Detailed N -glycan analysis of mouse serum on individual WAX fractions. (PDF) [file pone.0071159.s001.pdf]
