# Supplementary material for: Increase in Sialylation and Branching in the Mouse Serum N-glycome Correlates with Inflammation and Ovarian Tumour Progression
Source: PLoS One. 2013 Aug 30;8(8):e71159. doi: 10.1371/journal.pone.0071159 (PMC3758313; doi:10.1371/journal.pone.0071159)
Supplement: Figure S2 — Negative ion electrospray MS/MS spectrum of the triantennary glycan (phosphate adduct) showed that triantennary glycans are branched on the 6-antenna. (DOC) [file pone.0071159.s002.doc]

**Figure S2**: **Negative ion electrospray MS/MS spectrum of the triantennary glycan** (phosphate adduct) **showed that triantennary glycans are branched on the 6-antenna**.

Fragment ion nomenclature is according to that proposed by Domon and Costello . Symbols for the structural diagram are as defined in the footnote to Table 1. The branching pattern is defined by the D, [D-18]^-^ and [D-36]^-^ ions at *m/z* 1053, 1035 and 1017 respectively and the absence of an ion at *m/z* 831 (diagnostic for branching on the 3-antenna) [2].

**References**

1. Domon B, Costello CE (1988) A systematic nomenclature for carbohydrate fragmentations in FAB-MS/MS spectra of glycoconjugates. Glycoconj Journal 5: 397-409.

2. Harvey DJ, Royle L, Radcliffe CM, Rudd PM, Dwek RA (2008) Structural and quantitative analysis of N-linked glycans by matrix-assisted laser desorption ionization and negative ion nanospray mass spectrometry. Anal Biochem 376: 44-60.
