# Supplementary material for: Increase in Sialylation and Branching in the Mouse Serum N-glycome Correlates with Inflammation and Ovarian Tumour Progression
Source: PLoS One. 2013 Aug 30;8(8):e71159. doi: 10.1371/journal.pone.0071159 (PMC3758313; doi:10.1371/journal.pone.0071159)
Supplement: Figure S3 — Sialic acid speciation by DMB labelling shows mouse serum contains mostly N -glycolylneuraminic acid. (DOC) [file pone.0071159.s003.doc]

**Figure S3**: **Sialic acid speciation by DMB labelling shows mouse serum contains mostly *N*-glycolylneuraminic acid**

Gradient separation of a standard mixture of DMB labelled sialic acids on a Waters XBridge BEH C18 2.1×15 cm, 3.5μm column using 10mM ammonium formate pH4.4 and acetonitrile. Analysis of mice sera showed that they contained mostly *N*-glycolylneuraminic acid with a small proportions of *N*-acetylneuraminic acid, 5-glycolyl-9-acetylneuraminic acid and 5-7/8-9-triacetylneuraminic acid.
