# Supplementary material for: Increase in Sialylation and Branching in the Mouse Serum N-glycome Correlates with Inflammation and Ovarian Tumour Progression
Source: PLoS One. 2013 Aug 30;8(8):e71159. doi: 10.1371/journal.pone.0071159 (PMC3758313; doi:10.1371/journal.pone.0071159)
Supplement: Table S1 — Correlations of N -glycome and tumour volume. (DOC) [file pone.0071159.s004.doc]

**Table S1: Correlations of *N***-glycome and tumour volume

| Sample | HILIC | | | WAX-HPLC | | | | Total volume (mm3) |
| --- | --- | --- | --- | --- | --- | --- | --- | --- |
|  | Peak 10 | Peak 11 | Peak 12 | S2 | S3A | S3B | S4 |  |
| G1M2W7 | 39.48 | 13.24 | 6.82 | 63.45 | 13.42 | 12.09 | 4.29 | 8.52 |
| G1M3W7 | 39.07 | 13.73 | 7.04 | 65.72 | 12.60 | 11.09 | 3.71 | 8.93 |
| G1M4W7 | 38.37 | 15.64 | 6.35 | 67.16 | 12.12 | 10.13 | 3.17 | 0.57 |
| G1M5W7 | 32.12 | 11.05 | 13.40 | 53.96 | 12.81 | 20.95 | 7.23 | 8.59 |
| G2M1W7 | 40.87 | 13.30 | 6.92 | 68.24 | 11.61 | 10.08 | 3.24 | 15.71 |
| G2M2W7 | 40.03 | 11.52 | 7.18 | 65.99 | 13.23 | 10.33 | 3.77 | 5.04 |
| G2M4W7 | 40.20 | 13.20 | 6.69 | 67.10 | 12.83 | 9.71 | 3.43 | 10.88 |
| G2M5W7 | 40.49 | 14.36 | 6.77 | 68.55 | 11.22 | 10.31 | 3.04 | 21.63 |
| G3M1W7 | 36.60 | 14.04 | 7.39 | 64.80 | 13.12 | 11.25 | 4.25 | 10.20 |
| G3M2W7 | 37.73 | 15.04 | 6.47 | 66.66 | 13.09 | 10.04 | 3.48 | 4.02 |
| G3M3W7 | 40.17 | 15.75 | 6.58 | 68.28 | 11.80 | 10.11 | 3.43 | 38.73 |
| G3M4W7 | 36.42 | 8.13 | 4.55 | 62.36 | 14.26 | 11.49 | 3.58 | 42.31 |
| G3M5W7 | 40.87 | 12.42 | 7.19 | 64.60 | 13.10 | 11.76 | 3.88 | 8.76 |
| G4M1W7 | 41.47 | 13.98 | 5.65 | 68.79 | 11.46 | 9.79 | 2.68 | 7.40 |
| G4M2W7 | 40.57 | 12.86 | 6.85 | 68.21 | 10.72 | 10.21 | 3.39 | 14.74 |
| G4M3W7 | 40.22 | 14.47 | 5.60 | 69.46 | 11.25 | 8.57 | 2.68 | 26.22 |
| G4M5W7 | 40.47 | 14.20 | 5.92 | 69.10 | 11.79 | 8.74 | 2.61 | 34.86 |
| G5M1W7 | 37.45 | 11.62 | 9.32 | 61.67 | 13.28 | 13.73 | 4.99 | 75.99 |
| G5M2W7 | 37.26 | 11.79 | 7.79 | 62.72 | 14.38 | 11.17 | 4.55 | 98.22 |
| G5M3W7 | 38.14 | 13.18 | 8.32 | 64.74 | 12.14 | 12.48 | 4.10 | 78.92 |
| G5M4W7 | 38.34 | 12.26 | 8.06 | 62.42 | 13.41 | 12.60 | 4.82 | 70.30 |
| G5M5W7 | 36.20 | 9.82 | 9.67 | 56.94 | 13.89 | 16.26 | 6.38 | 141.01 |
| G6M1W7 | 39.75 | 14.04 | 7.05 | 68.62 | 10.45 | 10.83 | 3.19 | 47.85 |
| G6M3W7 | 38.65 | 15.66 | 6.49 | 67.69 | 10.18 | 11.48 | 3.10 | 5.72 |
| G6M4W7 | 39.12 | 14.27 | 6.13 | 66.55 | 12.59 | 9.74 | 3.41 | 13.48 |
| G6M5W7 | 39.45 | 15.56 | 5.75 | 68.43 | 12.10 | 9.05 | 3.08 | 55.14 |
| G7M1W7 | 40.87 | 13.85 | 6.01 | 69.07 | 11.74 | 8.96 | 2.84 | 0.00 |
| G7M2W7 | 41.98 | 11.80 | 5.89 | 67.38 | 12.33 | 9.51 | 3.02 | 0.00 |
| G7M3W7 | 41.60 | 15.78 | 5.29 | 71.40 | 10.31 | 8.99 | 2.14 | 0.00 |
| G7M4W7 | 39.28 | 11.11 | 6.92 | 65.63 | 13.20 | 9.94 | 4.01 | 0.00 |
| Association of peaks with tumour volumes | | | | | | | |  |
| Peak | Peak 10 | Peak 11 | Peak 12 | S2 | S3A | S3B | S4 |  |
| P-value | 0.039 | 0.049 | 0.038 | 0.009 | 0.042 | 0.025 | 0.007 |  |
| Pearson correlation coefficient | -0.379 | -0.379 | 0.381 | -0.468 | 0.373 | 0.409 | 0.483 |  |

G(x)=group number, M(x)=mouse number, W(x)=week number

Tumour volumes were measured in week 7
