# Supplementary material for: Increase in Sialylation and Branching in the Mouse Serum N-glycome Correlates with Inflammation and Ovarian Tumour Progression
Source: PLoS One. 2013 Aug 30;8(8):e71159. doi: 10.1371/journal.pone.0071159 (PMC3758313; doi:10.1371/journal.pone.0071159)
Supplement: Table S2 — Sialyltranferases with promoters conserved among species. (DOC) [file pone.0071159.s005.doc]

**Table S2:** Sialyltranferases with promoters conserved among species

| RefSeq accession number | Associated Gene Name | Description |
| --- | --- | --- |
| NM_003033 | ST3GAL1 | ST3 beta-galactoside alpha-2,3-sialyltransferase 1 [Source:HGNC Symbol;Acc:10862] |
| NM_173344 | ST3GAL1 | ST3 beta-galactoside alpha-2,3-sialyltransferase 1 [Source:HGNC Symbol;Acc:10862] |
| NM_006927 | ST3GAL2 | ST3 beta-galactoside alpha-2,3-sialyltransferase 2 [Source:HGNC Symbol;Acc:10863] |
| NM_006279 | ST3GAL3 | ST3 beta-galactoside alpha-2,3-sialyltransferase 3 [Source:HGNC Symbol;Acc:10866] |
| NM_003896 | ST3GAL5 | ST3 beta-galactoside alpha-2,3-sialyltransferase 5 [Source:HGNC Symbol;Acc:10872] |
| NM_006100 | ST3GAL6 | ST3 beta-galactoside alpha-2,3-sialyltransferase 6 [Source:HGNC Symbol;Acc:18080] |
| NM_173216 | ST6GAL1 | ST6 beta-galactosamide alpha-2,6-sialyltranferase 1 [Source:HGNC Symbol;Acc:10860] |
| NM_032528 | ST6GAL2 | ST6 beta-galactosamide alpha-2,6-sialyltranferase 2 [Source:HGNC Symbol;Acc:10861] |
| NM_018414 | ST6GALNAC1 | ST6 (alpha-N-acetyl-neuraminyl-2,3-beta-galactosyl-1,3)-N-acetylgalactosaminide alpha-2,6-sialyltransferase 1 [Source:HGNC Symbol;Acc:23614] |
| NM_006456 | ST6GALNAC2 | ST6 (alpha-N-acetyl-neuraminyl-2,3-beta-galactosyl-1,3)-N-acetylgalactosaminide alpha-2,6-sialyltransferase 2 [Source:HGNC Symbol;Acc:10867] |
| NM_152996 | ST6GALNAC3 | ST6 (alpha-N-acetyl-neuraminyl-2,3-beta-galactosyl-1,3)-N-acetylgalactosaminide alpha-2,6-sialyltransferase 3 [Source:HGNC Symbol;Acc:19343] |
| NM_030965 | ST6GALNAC5 | ST6 (alpha-N-acetyl-neuraminyl-2,3-beta-galactosyl-1,3)-N-acetylgalactosaminide alpha-2,6-sialyltransferase 5 [Source:HGNC Symbol;Acc:19342] |
| NM_003034 | ST8SIA1 | ST8 alpha-N-acetyl-neuraminide alpha-2,8-sialyltransferase 1 [Source:HGNC Symbol;Acc:10869] |
| NM_006011 | ST8SIA2 | ST8 alpha-N-acetyl-neuraminide alpha-2,8-sialyltransferase 2 [Source:HGNC Symbol;Acc:10870] |
| NM_015879 | ST8SIA3 | ST8 alpha-N-acetyl-neuraminide alpha-2,8-sialyltransferase 3 [Source:HGNC Symbol;Acc:14269] |
| NM_005668 | ST8SIA4 | ST8 alpha-N-acetyl-neuraminide alpha-2,8-sialyltransferase 4 [Source:HGNC Symbol;Acc:10871] |
| NM_013305 | ST8SIA5 | ST8 alpha-N-acetyl-neuraminide alpha-2,8-sialyltransferase 5 [Source:HGNC Symbol;Acc:17827] |
