# Supplementary material for: Increase in Sialylation and Branching in the Mouse Serum N-glycome Correlates with Inflammation and Ovarian Tumour Progression
Source: PLoS One. 2013 Aug 30;8(8):e71159. doi: 10.1371/journal.pone.0071159 (PMC3758313; doi:10.1371/journal.pone.0071159)
Supplement: Methods S1 — Supplementary Materials and Methods, Results, and Discussion. (DOC) [file pone.0071159.s006.doc]

**SUPPORTING INFORMATION**

**Supplementary Materials and Methods**

**Statistical analysis of promoter conservation of sialyltransferases**

The promoter sequences were taken from the cross species alignment of human, mouse, rat and dog promoters generated by Xie *et al.* . The information was based on the NCBI RefSeq project . The dataset is centered on the proximal promoter region, 2kb upstream and 2kb downstream of the RefSeq annotated transcription start site (TSS) for ~17,000 genes. A shorter promoter region was used that did not include the protein coding sequence, if the transcription start site was within 2kb of the RefSeq annotated TSS. Across all four species, it was possible to align ~51% of the sequences. The promoter conservation length was the number of bases within the human promoter that could be aligned with mouse, rat and dog. The figure was used as a basis of comparison between the sialyltransferases and the dataset as a whole. To assess the statistical significance of the results we used the non-parametric Mann-Whitney test (two tailed), using the R statistics package (http://cran.r-project.org/).

**Supplementary Results**

**Sialyltransferases alterations in the pre-clinical mouse model are relevant to other species**

To evaluate whether these results are relevant to the human situation, we analysed gene promoter conservations focusing on the proximal promoter regions of the sialyltransferase genes (4kb upstream of the start of transcription). We detected a significantly greater degree of conservation in the promoters of the 17 sialyltransferases (Supplementary Table S2), including these responsible for the glycan changes described in detail above, when compared to the background set of human promoters (~17,000 promoters, p=0.023). This was based on the proportion of bases within the human proximal promoters that could be aligned with mouse, rat and dog sequences. The median conservation length per bp was 0.545bp for promoters as a whole compared to 0.79bp for sialyltransferases.

We also critically analysed the differences between mouse and human oligosaccharides to place this information in the context of altered liver enzyme expression, in both human and mouse species. In comparison with human serum, mouse serum contained galactose linked both β1-3 and β1-4 (Table 1), human serum has all galactose residues linked β1-4 to GlcNAc . Sialic acids are mostly Neu5Gc in mouse (Supplementary Figure S3) and Neu5Ac in human . In mouse, some sialic acids are also linked to GlcNAc, creating more sialylation than branches (Table 1) . In human serum glycoproteins, the sialic acids are linked to Gal and are always less than or equal to the number of branches . No outer arm fucosylation or bisected GlcNAc residues were present on mouse glycans (Table 1). In contrast, these are present on human glycans .

In spite of these differences, our findings of increased sialylation in the presence of tumours and pro-inflammatory compounds would still be relevant to cancer patients. This is because data mining of the promoter regions of the sialyltransferases was consistent with conserved liver expression of the enzymes in human and mouse.

**Supplementary Discussion**

The bioinformatic data mining of promoter sequence conservation over several species suggests that sialyltransferase genes appear to be more tightly regulated, than genes as a whole, as is evident by the greater degree of conservation in their promoter regions. There are greater evolutionary constraints on the promoters of these genes than one would expect by chance. This implies that there is a high degree of selective pressure to maintain these regulatory regions, suggesting that these genes perform similar functions across the species included in this analysis. Therefore, we suggest that the described increase in sialylation with tumour progression and pro-inflammatory stimuli would also affect humans. Indeed, increase in sialylation and branching was reported in human inflammation and cancer and MGAT5 has previously been associated with metastasis ,. Increased branching creates more sites for the addition of terminal sialic acid residues and together with upregulation of sialyltransferase results in increased sialylation . Increased expression of ST6Gal1 which is responsible for adding α2-6-linked sialic acid on *N*-glycans mediates tumour progression and causes a less differentiated phenotype *via* enhanced β1-integrin function . Sialylation is associated with cancer progression .

**Supplementary References**

1. Xie X, Lu J, Kulbokas EJ, Golub TR, Mootha V, et al. (2005) Systematic discovery of regulatory motifs in human promoters and 3' UTRs by comparison of several mammals. Nature 434: 338-345.

2. Pruitt KD, Maglott DR (2001) RefSeq and LocusLink: NCBI gene-centered resources. Nucleic Acids Res 29: 137-140.

3. Royle L, Campbell MP, Radcliffe CM, White DM, Harvey DJ, et al. (2008) HPLC-based analysis of serum N-glycans on a 96-well plate platform with dedicated database software. Anal Biochem 376: 1-12.

4. Lin SY, Chen YY, Fan YY, Lin CW, Chen ST, et al. (2008) Precise mapping of increased sialylation pattern and the expression of acute phase proteins accompanying murine tumor progression in BALB/c mouse by integrated sera proteomics and glycomics. J Proteome Res 7: 3293-3303.

5. Coddeville B, Regoeczi E, Strecker G, Plancke Y, Spik G (2000) Structural analysis of trisialylated biantennary glycans isolated from mouse serum transferrin. Characterization of the sequence Neu5Gc(alpha 2-3)Gal(beta 1-3)[Neu5Gc(alpha 2-6)]GlcNAc(beta 1-2)Man. Biochim Biophys Acta 1475: 321-328.

6. Arnold JN, Saldova R, Hamid UM, Rudd PM (2008) Evaluation of the serum N-linked glycome for the diagnosis of cancer and chronic inflammation. Proteomics 8: 3284-3293.

7. Dennis JW, Laferte S, Waghorne C, Breitman ML, Kerbel RS (1987) Beta 1-6 branching of Asn-linked oligosaccharides is directly associated with metastasis. Science 236: 582-585.

8. Kim YJ, Varki A (1997) Perspectives on the significance of altered glycosylation of glycoproteins in cancer. Glycoconj J 14: 569-576.

9. Hedlund M, Ng E, Varki A, Varki NM (2008) alpha 2-6-Linked sialic acids on N-glycans modulate carcinoma differentiation *in vivo*. Cancer Res 68: 388-394.

10. Miyagi T (2008) Aberrant expression of sialidase and cancer progression. Proc Jpn Acad Ser B Phys Biol Sci 84: 407-418.
